# To respond or not to respond? Natural variation of root architectural responses to nutrient signals

**DOI:** 10.1093/jxb/erx160

**Published:** 2017-07-17

**Authors:** Anna Amtmann, Zaigham Shahzad

**Affiliations:** Institute of Molecular, Cellular and Systems Biology, College of Medical, Veterinary, and Life Sciences, University of Glasgow, Glasgow, UK

**Keywords:** Environmental interactions, epistatic effects, glutamate, natural variation, nitrate, QTL mapping, root architecture, root growth, temperature sensitivity

## Abstract

This article comments on:

Walch-Liu P, Meyer RC, Altmann T, Forde BG. 2017. QTL analysis of the developmental response to L-glutamate in Arabidopsis roots and its genotype-by-environment interactions. Journal of Experimental Botany 68, 2919–2931.

**The amino acid glutamate (Glu) acts as a fast excitatory neurotransmitter in mammals. Its importance in plant signalling was recognized with the discovery of channel proteins similar to mammalian Glu receptors, as well as distinct changes in root-system architecture in response to very small amounts of soil Glu. Based on natural genetic variation within Arabidopsis, Walch-Liu *et al.* (2017) have now identified a major locus underpinning this root response, as well as several loci controlling it through gene by environment interactions with nitrate and temperature. It is a significant step towards unraveling crosstalk between signalling pathways that enable plants to adjust their growth and development to multiple environmental stimuli.**

In order to survive as a sessile organism in a given environment a plant needs to adjust its growth and development to environmental factors such as light and temperature and the availability of water and mineral nutrients. They therefore possess a sensory system of receptors and downstream signalling components that is at least as sophisticated as animal neuronal networks. Furthermore, despite the lack of a central brain, plants are able to integrate many different stimuli and make informed decisions about which responses to prioritize. The decision-making process is guided by molecular hubs that enable crosstalk between individual signalling pathways, and we are now starting to unravel the precise wiring of this complex network. For example, a recent study shows that the key transcription factor PHR1, which regulates the phosphate stress response in Arabidopsis, also directly represses defence (Castrillo *et al.*, 2017), thus demonstrating that the plant prioritizes nutritional stress responses over defence.

## Plant nitrogen sources

The uptake of nitrogen (N) as a building block for proteins is one of the central functions of plant roots. The uptake of N has to be adjusted to the rate of carbon assimilation through the leaves, which depends on light, CO_2_ levels, humidity and temperature, and accommodated with other root functions such as anchorage and water uptake, which depend on soil structure and soil water profiles. Plants often alter the growth and spatial arrangement of the root system (root-system architecture, RSA) in response to edaphic cues in order to explore the soil for available resources (Drew, 1975; Kellermeier *et al.*, 2013). Inorganic nitrate (NO_3_^–^) is the preferred source of N, and the molecular entities underpinning nitrate perception, regulation of nitrate transporters and adjustment of RSA to nitrate availability have been studied in great detail (Ho *et al.*, 2009; Forde, 2014*a*). However, in soils with low rates of N mineralization, plants can also take advantage of organic N present in the soil (Schimel and Bennett, 2004; Rothstein, 2009). Amino acids are good candidates for reporting on organic N as they are used as chemical foraging cues by a wide variety of motile organisms (Lux and Shi, 2004).

## Glutamate acts as a signal in plants

At high (1–10 mM) concentrations most amino acids inhibit plant cell growth. The inhibitory effects of most of them can be overcome by equimolar supply of glutamine (Gln), and is therefore likely to be due to relative Gln deficiency (Bonner *et al.*, 1996). However, Forde (2014*b*) reported that several amino acids significantly inhibit primary root growth of Arabidopsis even in the presence of Gln. Among these, Glu is the only one that also stimulates secondary root growth. A potential signalling role of Glu had previously been proposed because inhibition of primary root growth can be elicited by very low (50 µM) exogenous concentrations localized around the root tip (Walch-Liu *et al.*, 2006). The Glu signal causes an initial slow-down of cell divisions in the primary root meristem followed by complete cessation of primary root growth and stimulation of secondary root emergence and growth. It is thought that the resulting short and branched RSA facilitates soil foraging. Interestingly, the Glu response can be prevented by simultaneous application of nitrate to the root tip suggesting that a foraging response to organic N is actively suppressed if there is enough inorganic N (Walch-Liu and Forde, 2008).

The notion that Glu acts as a signal in plants (Forde, 2014*b*) was in line with claims that Glu receptor-like ion channels (GLRs) homologous to mammalian ionotropic glutamate receptors (iGluRs) could have important functions in the plant sensory system (Chiu *et al.*, 1999; Davenport, 2002). A clear link between these channels and Glu signalling has not yet been established, but one study showed that a rice GLR is critical for cell division in the root apical meristem (Li *et al.*, 2006). A recent chemical genetics study aimed at discovering proteins that are necessary for the root Glu response of Arabidopsis also failed to identify GLRs (Forde *et al.*, 2013). Instead, the screen revealed MEKK1, a MAP kinase kinase kinase, as a positive regulator of Glu signalling. This finding nevertheless points to analogy between plant and mammalian Glu signalling as the MAP kinase pathways play an important role in the iGluR-mediated response to L-Glu in mammals (Wang *et al.*, 2007).

## Genetic loci underpin Glu sensitivity and its modulation by other factors

Arabidopsis accessions vary considerably in their root response to Glu (Walch-Liu *et al.*, 2006): for example, C24 is very sensitive whereas Col-0 is rather insensitive to Glu. Walch-Liu *et al.* (2017) have now exploited this natural variation to identify the genetic basis of Glu signalling. They measured the primary root length of several hundred recombinant inbred lines from reciprocal Col-0 × C24 crosses, before and after transfer of young seedlings onto Glu-containing media. QTL analysis identified one major and two additional loci which together explain 40% of the variation, and using additional introgression lines they were able to narrow down the former to a region containing some 200 genes. One of the genes encodes a MAPK, which is now a good candidate for a novel Glu-signalling component.

Where this research goes further than most other QTL studies of RSA nutrient responses is investigation of the genetic basis for the modulation of the Glu response by other factors, in particular nitrate, which suppressed the Glu response to various extents in the lines, and temperature, which in a relatively moderate range between 20 and 26 **°**C also altered the response. This multifactorial experimental design allowed them to discover ‘ectopic’ loci underpinning the nitrate and temperature dependence of Glu signalling, thereby supporting the notion that combinatorial environmental treatments greatly enhance the power of natural variation studies (Tonsor *et al.*, 2005). The results now provide an excellent basis for identifying the genes that enable crosstalk between Glu, nitrate and temperature signalling (Box 1).

**Box 1. QTLs underlying the glutamate response of Arabidopsis and its dependence on nitrate and temperature**

Some Arabidopsis genotypes respond to glutamate (Glu) with a cessation of primary root growth and subsequent initiation of long laterals just above the root tip. The response is suppressed by nitrate and modulated by temperature (Temp). The strength of the response varies among accessions: for example, C24 shows a strong response whereas Col-0 is less sensitive to Glu. Walch-Liu *et al.* (2017) used the natural variation of this response in a Col-0 × C24 inbred population to identify several quantitative trait loci (QTLs) in the Arabidopsis genome. The Glu-sensitivity loci *GluS1–3* together explain 40% of variation of the Glu response. *GluS4* and *GluS5* also contribute to Glu sensitivity but only under certain conditions of temperature or nitrate. *NS1* and *TS1,2* are additional independent loci that modulate the sensitivity of the Glu response to nitrate or temperature, respectively.

**Figure:**
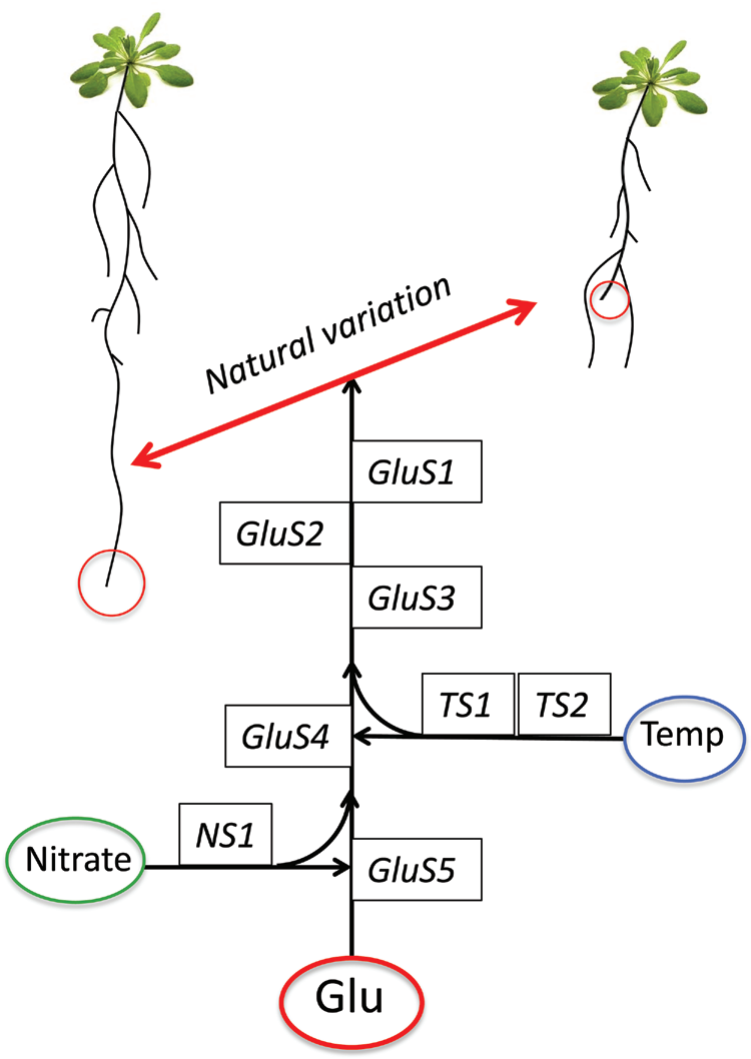

## Natural variation allows evaluation of physiological consequences of RSA responses

Despite successful examples of QTL-based discovery of genes underpinning root responses (Svistoonoff *et al.*, 2007; Pineau *et al.*, 2012; Hu *et al.*, 2015; Shahzad *et al.*, 2016), the sad truth is that discovery of quantitative loci has not always led to gene identification. The fact that the loci identified by Walch-Liu *et al.* (2017) are relatively small and that the QTL effects are relatively large is promising. The authors have already highlighted several candidates within the loci based on previous knowledge of Glu signalling but, even if these turn out to be wrong, identification of the ‘culprits’ through further fine mapping and sequencing combined with knockout mutants seems a manageable task and there is hope for the future.

So should we take notice of QTL studies in which the underpinning genes have not yet been positionally cloned? The answer is yes, because identification of new genes is only one of the informative values of natural variation. Similarly important is that natural variation allows us to evaluate the physiological consequences of the different responses and the environmental constraints that limit their benefits (Box 2). As explained above, the foraging theory provides a reasonable explanation as to why an RSA response to Glu, and its integration with other environmental cues, could provide a benefit to the plant. While this makes perfect sense, it leads immediately to the question as to why some genotypes do *not* respond to the stimulus. We would argue that addressing this question is absolutely necessary if we want to effectively translate the genetic information gained form QTL studies to agriculture. Each of the possible answers to the ‘why-not respond’ question has interesting implications. Answer 1: it doesn’t matter. While generally assumed, there is in fact very little hard evidence that reported RSA changes to nutritional signals are beneficial. What we urgently need are physiological studies that investigate whether a particular RSA enhances nutrient uptake or not. Recent studies on maize genotypes have provided the first evidence that certain root shapes are beneficial for plant growth on low phosphate and low nitrate (Postma *et al.*, 2014; Zhan and Lynch, 2015), but a lot more research is needed to prove the ‘nutrient foraging’ hypothesis for other nutrients and plant species. Preliminary studies with Arabidopsis accessions in our laboratory indicate that in some cases genotypes with smaller roots compensate for this feature with higher nutrient uptake per root surface. It is important to consider this possibility to prevent disappointment when trying to improve nutrient uptake in crops through altered root architecture. Clearly, further investigations are needed into the consequences of RSA responses for nutrient uptake, and for this we need experimental systems that allow us not only to apply precise nutrient concentrations to the roots but also to generate defined nutrient profiles. The identification of natural genotypes or near-isogenic lines that strongly differ in RSA responses to different nutrients provides us with a wonderful resource to embark on such studies.

**Box 2. Maximal information gain from natural variation**

Starting from natural variation of root architectural responses to glutamate (Glu), we can identify the genetic components of the underlying signalling network, study the physiological consequences of the phenotypes, and correlate genotypes with natural environmental factors. Combining these different pieces of information can guide the development of crop varieties with improved nitrogen use efficiency and maximize their benefit in different field scenarios.

**Figure:**
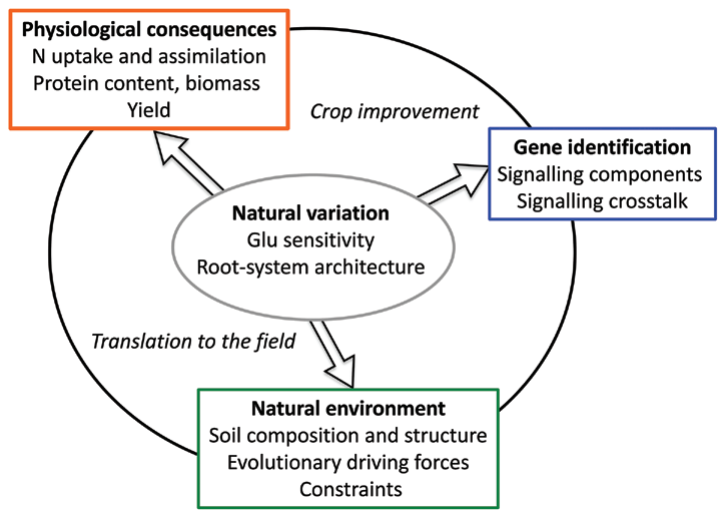

## Natural environment and environmental constraints determine root responsiveness

If answer 1 is wrong, and root architectural changes are indeed beneficial for nutrient uptake, we need to look to the environment in which the plants have evolved. Assuming that the non-responsive genotypes are not stupid – and nature rarely is stupid! – it is likely that they simply don’t experience the stimulus in nature. For example, Glu-insensitive lines may have evolved in nitrate-rich soils. Walch-Liu and colleagues allude to the role of the natural environment when pointing out that the C24 accession, which is least temperature sensitive, originates from the most southerly latitude. As widely lamented, most of our current collections of Arabidopsis accessions are not suitable for further investigating this issue, as there is insufficient metadata for the environments in which they have been collected. Some research groups have already collected new local accessions together with precise environmental information in order to answer questions related to flowering and temperature (Sasaki *et al.*, 2015) or salinity (Busoms *et al.*, 2015), and plant nutritionists will need to do the same, joining hands with soil scientists to obtain precise information on nutrient profiles.

Answer 2 to the ‘why-not respond’ question is that an RSA response would be beneficial for the non-response genotypes in their natural habitats, but that there are other environmental constraints that have precluded their evolution. Again, precise knowledge of such constraints and their physiological consequences is paramount for knowledge transfer to the field. Particular emphasis should be put on soil structural features that may either limit how much the root system can expand, or demand prioritization of RSAs that ensure effective anchoring or water uptake. Toxicity should also be considered as some nutrient-rich patches may also contain high amounts of heavy metals or, in the case of organic nitrogen, microbial toxins. Research into interactive effects of different environmental factors has recently gained momentum and we can expect revolutionary knowledge gain from controlled multi-factorial studies. The findings of Walch-Liu and colleagues that nitrate and temperature modulate the Glu response are highly meaningful in this context. Similarly exciting would be to discover factors that can release a putative ‘response block’ in the insensitive lines.

In summary, natural variation offers us an excellent tool to identify the genetic entities that underpin nutrient signalling and its integration with other signalling pathways. However, even if the genes are not (yet) identified, the informative value of natural variation is enormous as it provides a basis for physiological and environmental studies that in the long term might prove even more important for crop improvement than the molecular knowledge.
